# Isolating Specific vs. Non-Specific Binding Responses in Conducting Polymer Biosensors for Bio-Fingerprinting

**DOI:** 10.3390/s21196335

**Published:** 2021-09-22

**Authors:** Phil M. Smith, Indorica Sutradhar, Maxwell Telmer, Rishikesh Magar, Amir Barati Farimani, B. Reeja-Jayan

**Affiliations:** 1 Department of Mechanical Engineering, Carnegie Mellon University, Pittsburgh, PA 15213, USA; smithpm89@gmail.com (P.M.S.); mrt@andrew.cmu.edu (M.T.); rmagar@andrew.cmu.edu (R.M.); barati@cmu.edu (A.B.F.); 2 Department of Materials Science & Engineering & Biomedical Engineering, Carnegie Mellon University, Pittsburgh, PA 15213, USA; indorica18@gmail.com

**Keywords:** vapor-phase polymerization (VPP), conducting polymers, chemiresistive biosensors, machine learning

## Abstract

A longstanding challenge for accurate sensing of biomolecules such as proteins concerns specifically detecting a target analyte in a complex sample (e.g., food) without suffering from nonspecific binding or interactions from the target itself or other analytes present in the sample. Every sensor suffers from this fundamental drawback, which limits its sensitivity, specificity, and longevity. Existing efforts to improve signal-to-noise ratio involve introducing additional steps to reduce nonspecific binding, which increases the cost of the sensor. Conducting polymer-based chemiresistive biosensors can be mechanically flexible, are inexpensive, label-free, and capable of detecting specific biomolecules in complex samples without purification steps, making them very versatile. In this paper, a poly (3,4-ethylenedioxyphene) (PEDOT) and poly (3-thiopheneethanol) (3TE) interpenetrating network on polypropylene–cellulose fabric is used as a platform for a chemiresistive biosensor, and the specific and nonspecific binding events are studied using the Biotin/Avidin and Gliadin/G12-specific complementary binding pairs. We observed that specific binding between these pairs results in a negative Δ*R* with the addition of the analyte and this response increases with increasing analyte concentration. Nonspecific binding was found to have the opposite response, a positive Δ*R* upon the addition of analyte was seen in nonspecific binding cases. We further demonstrate the ability of the sensor to detect a targeted protein in a dual-protein analyte solution. The machine-learning classifier, random forest, predicted the presence of Biotin with 75% accuracy in dual-analyte solutions. This capability of distinguishing between specific and nonspecific binding can be a step towards solving the problem of false positives or false negatives to which all biosensors are susceptible.

## 1. Introduction

Due to the ever-growing need to detect different biomolecules such as proteins or enzymes, the advancement of biosensing technology has become an interdisciplinary area of research, bringing together biologists, physicists, chemists, and engineers. The medical field, food industry, and environmental monitoring are a few areas where biosensors are used [1,2,3]. A typical biosensor consists of the detected analyte, a bioreceptor which binds specifically to the detected analyte, and a transducer that converts the binding event into a measurable signal [4]. Sensing platforms such as enzyme-linked immunosorbent assay (ELISA) and surface plasmon resonance (SPR) are based on immunoassays and plasmon generation, respectively. However, ELISA suffers from a relatively long sample preparation and high costs, which limits it to laboratory applications [5] and SPR suffers from low sensitivity to low molecular weight molecules since it is mass-sensitive [6].

All these platforms work on the principle that sensing occurs when a targeted analyte specifically attaches to a capture molecule anchored on a substrate. However, exposure to other non-targeted analytes for example, in complex samples such as blood, will result in nonspecific binding events, producing a signal that obscures the signal from the analyte of interest, essentially adding noise to the measurement. Nonspecific binding also occurs when the targeted analyte binds to sites other than the capture molecule. Every sensing platform suffers from this fundamental drawback, which limits its sensitivity, specificity, and longevity. Existing efforts to improve signal-to-noise ratio involve introducing steps to reduce nonspecific binding by imposing a blocking layer to shield unoccupied binding sites [7,8]. This blocking layer reduces analyte adsorption onto the unoccupied sites without interfering with the capture molecule and targeted analyte chemistries. Three main types of blocking agents used are detergent blockers, protein blockers, and polymer-based blockers, each one with their own advantages and disadvantages [9,10]. Another way to improve signal to noise is to engineer materials for a sensing platform capable of detecting/distinguishing between the two binding events.

Materials that exhibit a change in electrical response due to a change in chemical environment are termed chemiresistors and sensors which use these materials as transducers are called chemiresistive sensors. These sensors are label-free, highly sensitive and require little to no sample preparation time [11,12,13]. Specifically, chemiresistive biosensors made from conducting polymers are low cost, operate at room temperature, and can be made flexible due to the non-brittle nature of polymers. The signal-to-noise ratio in conducting polymer sensors can be improved by a higher conducting polymer film. This also provides an increase in the sensitivity of the sensor [14]. Another advantage of using conducting polymers is being able to modify the sensor surface with receptors to specifically target certain molecules while keeping the sensor platform the same. Poly (3,4-ethylenedioxyphene) (PEDOT) is one of the most widely used conducting polymers due to its optical transparency, mechanical flexibility, high electrical conductivity, and chemical and physical stability. It is typically copolymerized with other polymers to harness their functional groups for attaching different capture molecules. PEDOT-based sensors have been used in the detection of a selective ligand for human influenza A virus (H1N1) by copolymerizing the monomer EDOT with another EDOT bearing oxylamine group. This served as a unit for introducing sialyllactose to the side chain of the copolymer which was used in the detection of H1N1 [15]. Similarly, the copolymer, PEDOT and poly (3-thiopheneethanol) (P3TE), deposited on a high surface area electro-spun nylon fiber mat, was used to immobilize avidin for the detection of Biotin [14]. Using the high surface area substrate, a 6-fold increase in sensor response time was observed as opposed to a flat substrate.

In this work, our sensor architecture is similar to the sensor prepared by Bhattacharyya et al. [14]. We, however, use vapor-phase polymerization (VPP) to deposit the polymer layer onto a high surface area polypropylene–cellulose fabric. This layer is made from the copolymerization of EDOT and 3TE into an interpenetrating network (IPN) of PEDOT and P3TE [14]. Iron (III) p-toluenesulfonate (Fe(PTS)_3_) is used to polymerize EDOT, while P3TE weaves through the initially deposited PEDOT to form an IPN. This IPN structure increases interfacial area, allowing for increased binding of the analyte capture molecules. This in turn increases the sensitivity of the device. Due to their high affinity for each other, the biomolecule pair of Biotin and Avidin are chosen as the test analyte and capture molecules, respectively, to observe specific binding events. Gliadin, a protein found in wheat and Casein, a protein found in milk are chosen to study nonspecific binding events. This work goes a step further using machine learning to decouple signals from specific and nonspecific binding events. Classification techniques are applied to the measured response of the sensor and predicted the presence of the test analyte (Biotin) with accuracies up to 75%.

## 2. Materials and Methods

### 2.1. Reagents and Materials

3,4-Ethylenedioxythiophene (EDOT) (97%), 3-thiopheneethanol (3TE) (98%), Iron(III) p-toluenesulfonate hexahydrate (Fe(PTS)_3_), (3-Glycidyloxypropyl)trimethoxysilane (GOPS), phosphate buffer solution (PBS) and α-Casein were obtained from Sigma-Aldrich. Bovine serum albumin (BSA), avidin and Biotin were obtained from Thermo Fisher Scientific. Gliadin was received from the Prolamin Work Group (PWG). All chemicals were used as received.

### 2.2. Vapor-Phase Polymerization of P(EDOT-3TE)

**Figure 1:**
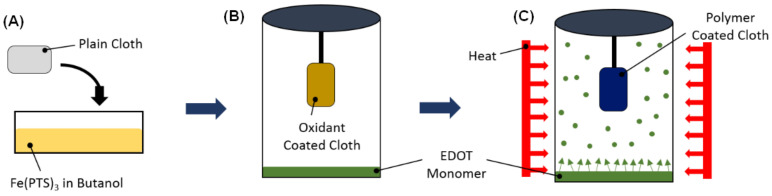
Schematic of vapor-phase polymerization (VPP) process for synthesis of PEDOT films on polypropylene–cellulose fabric. (**A**) The oxidant solution is a mixture of iron(III) p-toluenesulfonate hexahydrate (Fe(PTS)_3_) in butanol. The fabric is coated by soaking in the oxidant solution. (**B**) The oxidant coated fabric is placed in a sealed container with the monomer. (**C**) Heating the container in a furnace causes the monomer to vaporize and polymerization occurs on the fabric.

The VPP technique used here for preparing the polymer films has been described in detail in a previous report [16]. A schematic of the VPP process is shown in Figure 1. A polypropylene–cellulose fabric was first soaked in a 40 wt.% solution of Fe(PTS)_3_ in butanol. This oxidant coated fabric was then placed in a sealed jar containing the monomer EDOT. The EDOT polymerization occurred in a furnace at 70 °C for 1 h. The PEDOT coated fabric was then rinsed in ethanol for 1 h to remove any unreacted monomer and oxidant. The fabric was then placed in another sealed jar containing 3TE and again polymerization occurred at 70 °C for 1 h.

### 2.3. Anchoring Avidin to the Sensor

Avidin is covalently attached to the polymer coated fabric via the linker molecule GOPS. A total of 50 µL of GOPS was placed in a sealed container along with the coated fabric at 120 °C for 2 h. A soak in ethanol for 1 h removed any excess GOPS molecules. Two subsequent washes in a 1:1 ratio of BSA to PBS was done for an hour each to minimize protein adsorption onto unoccupied binding sites of the sensor surface. The attachment of avidin took place overnight in a 10 mL PBS solution with 1 mg avidin. The coated fabric was then soaked in a pure PBS solution for 10 min to remove unattached avidin molecules. Finally, the completed sensor was stored in a pure PBS solution. This was also the environment in which the testing took place.

### 2.4. Characterization

The VPP polymer films were characterized by Fourier transform infrared (FTIR) spectroscopy using a Thermo Fisher Scientific (Waltham, MA, USA) iS50 spectrometer. Scanning electron microscopy (SEM) images were recorded using a FEI Quanta 600 FEG SEM. The response of the sensor was measured using a biologic Sp-150 potentiostat.

### 2.5. Resistance Measurement of Sensor

After the avidin attachment, the sensors were submerged in PBS and alligator clips were then attached. The measurements were done by adding different analytes which included plain PBS and different concentrations of biomolecules in PBS to the container which housed the submerged sensor. To evaluate the response of the sensors a constant DC current of 950 µA was used. The resistance was monitored over a span of 30 min with the first 15 min being the time necessary for the steady state resistance to be attained then at the 15 min mark the analyte is added. The percent change in resistance is given by

(1) $$\Delta R\left(\%\right)=\frac{R_{0}-R_{1}}{R_{1}}\times 100,$$

where *R*_1_ is the resistance before the analyte is added (at 15 min) and *R*_0_ is the resistance at the end of the experiment (after 30 min). Two analytes, Biotin and Gliadin were tested at concentrations of 50 µM, 5 µM, 500 nM, and 50 nM.

### 2.6. Machine Learning

The first step in our approach is the exploratory data analysis (EDA). In the EDA step, the variation of voltage with time was carefully observed and features were extracted manually from the dataset. Signal processing techniques were also applied to extract information such as peak-to-peak difference (difference between minimum and maximum values of voltage), max signal (maximum voltage) and Kurtosis. These features were used as input to predict the presence of Biotin. The data considered in this analysis comprised of the voltage signal after the analyte was added.

Four common machine-learning algorithms were used to evaluate classification accuracies: support vector machines (SVM), random forest (RF), K-nearest neighbors (KNN) and logistic regression (LR). SVM perform well on the high-dimensional data and are less prone to overfitting. However, it does not offer interpretability from the algorithm. Optimizing the hyperparameters that give the best performance is a difficult task. RF is capable of reduction in overfitting as it aggregates information from an ensemble of decision trees. This algorithm is suitable for all types of data. However, it is computationally expensive. LR is easier to implement, interpret, and very efficient to train. However, it constructs linear boundaries and fails in cases where there is a nonlinear boundary. The model is prone to overfitting. Finally, KNN is computationally inexpensive and easier to train but does not work very well with large datasets and high-dimensional data.

Python, with the Scikit-learn package was used to code the SVM, RF, KNN, and LR. The dataset was split into 80% training data and 20% test set data on which the predictions were made. As a result, the total 73 data points were split into 58 train and 15 test datapoints. A 5-fold cross validation was done to minimize the variance ensuring that model accuracies obtained were more reliable.

## 3. Results and Discussion

**Figure 2:**
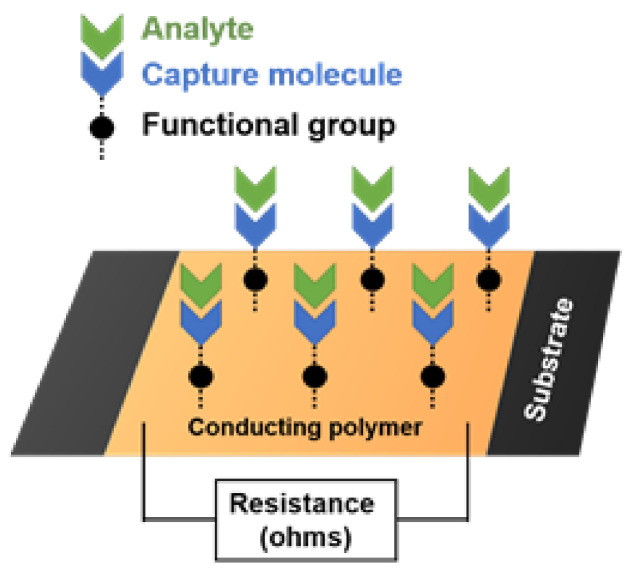
Schematic of chemiresistive sensor with capture molecule immobilization and analyte binding.

**Figure 3:**
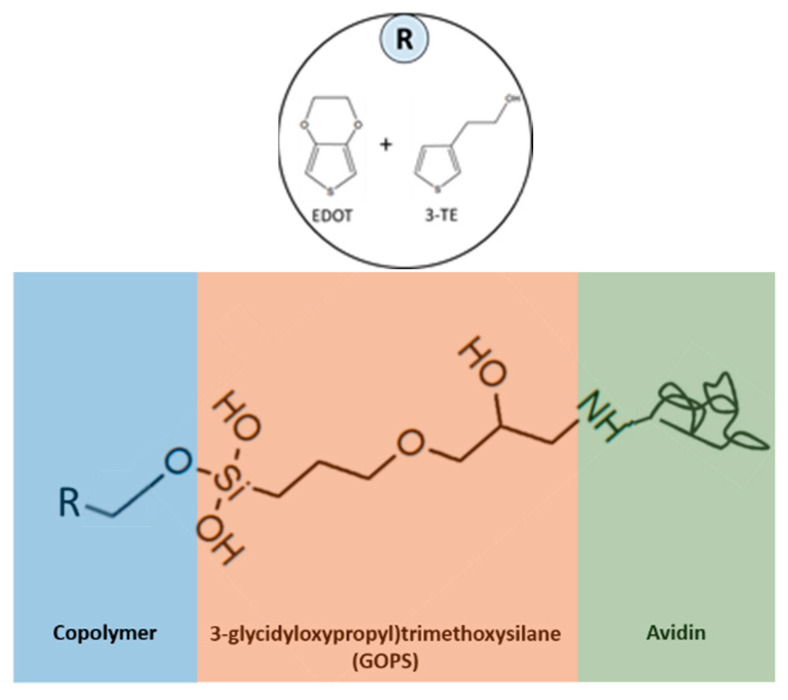
Avidin immobilization onto conducting polymer.

In the VPP process, an oxidant in the liquid phase is directly deposited onto the fabric substrate. It is then exposed to the monomer vapor in a sealed container where the polymerization of the conducting polymer occurs. Typical oxidants used in this process are iron(III)chloride, copper(II)chloride and iron (III) PTS. Iron (III) PTS was chosen here because PEDOT films produced with this oxidant has the highest reported electrical conductivity of ~1300 S/cm [17,18]. A schematic of the device structure is shown in Figure 2. The capture molecules are immobilized onto the polymer surface by the functional groups of the polymer and the analyte binding to the capture molecules occurs because they have a strong affinity for each other. To immobilize the molecules responsible for capturing the analyte, linker molecules must be first bound to the conducting polymer surface. Due to the lack of appropriate functional groups, the capture molecule, in this case avidin, cannot bind directly to the polymer surface. Linker molecules such as GOPS act as a bridge between the polymer layer and the capture molecule. Figure 3 shows the steps taken for attachment. GOPS is a silane coupling agent which contains two different reactive groups bonded to the silicon molecule. One end of this molecule is the methoxy group while the other end is the epoxy group. The methoxy group binds with the hydroxyl group from the P3TE while the epoxy group binds to the capture molecule.

In this work, biomolecule pairs with a high affinity for each other were chosen to test the response of the sensor. The first pair studied was the protein pair avidin and Biotin. Avidin is a protein typically found in the egg whites while Biotin is a B vitamin. The large protein avidin, can bind 4 of the small Biotin molecules with dissociation constant on the order 10^−8^s^−1^ of making it one of the strongest non-covalent bonds [19,20]. This property of the avidin/Biotin pair makes them popular in Western blotting [21] and in immunoassays such as ELISA [22]. The other biomolecule pair is the antigen-antibody pair of G12 and Gliadin. Gliadin is a class of protein present in gluten (protein found in wheat) known to trigger reactions in Celiac patients, and G12 is its respective antibody. The G12/Gliadin pair has also been applied in immunoassays such as ELISA [23].

**Figure 4:**
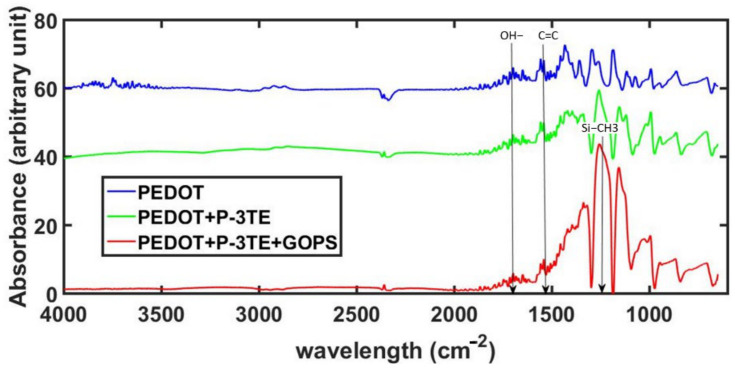
FTIR spectrum of PEDOT, PEDOT + P3TE and PEDOT + P3TE + GOPS measured on a silicon wafer.

FTIR was performed to check for successful growth of PEDOT and P3TE, as well as successful attachment of the GOPS molecule. FTIR was performed using a silicon wafer as the substrate instead of the fabric. The polymer growth and GOPS attachment processes on the silicon wafer were identical to processes done on the fabric. Figure 4 shows the spectrum of plain PEDOT, PEDOT + P3TE and PEDOT + P3TE + GOPS. The peak correlating with the formation of the C=C in PEDOT is seen at about 1517 cm^−1^ indicating the successful polymerization of EDOT. The addition of 3TE introduces –OH functional groups which are captured in the spectrum at ~3400 cm^−1^. Additionally, the detection of silicon (~1150 cm^−1^) indicates the presence/successful attachment of GOPS to the interpenetrating polymer network of PEDOT and P3TE.

**Figure 5:**
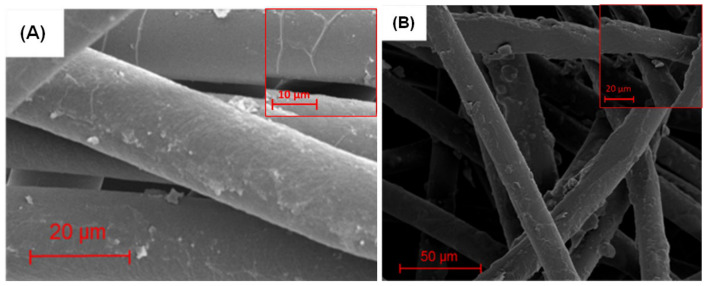
Scanning electron microscopy (SEM) of (**A**) uncoated fabric (**B**) fabric coated with PEDOT.

SEM images of the fabric substrate before and after PEDOT growth are shown in Figure 5. Comparing the two images, the individual fibers of the fabric seen in Figure 5B appear rougher than the ones of the uncoated fabric indicating the presence of PEDOT. It should also be noted that the morphology of the underlying fibers is retained, and that little aggregation of the polymer is observed as is commonly seen when solutions are present. The retention of the fiber morphology is important because the surface to volume ratio is increased, creating more available binding sites for the analyte, increasing the ability of the sensor to detect low concentrations of the analyte.

**Figure 6:**
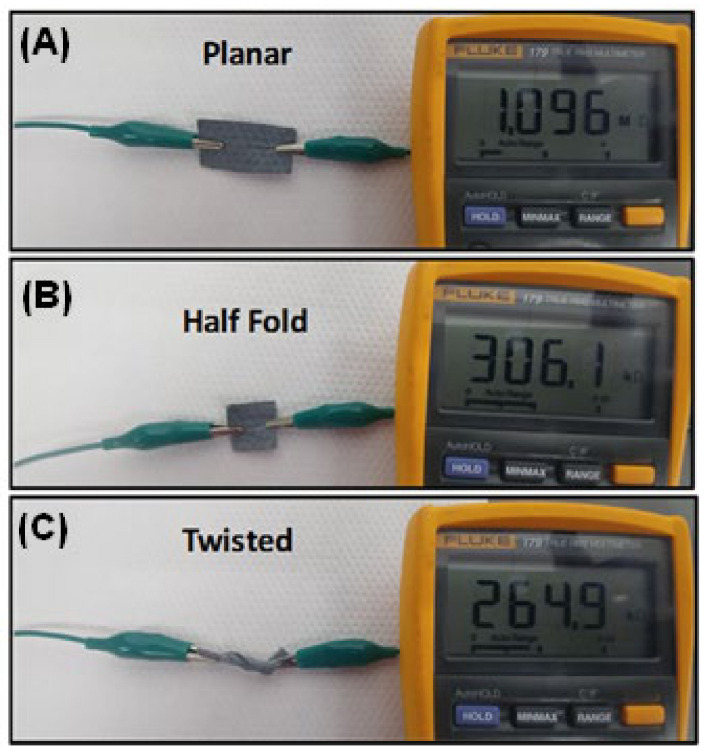
Polymer coated fabric in (**A**) planar: 1.096 MΩ (**B**) half fold: 306.1 kΩ and (**C**) twisted conformations: 264.9 kΩ.

As a side note, using a polymer-based sensor, we have the added benefit of having a low cost and mechanically flexible sensor [24]. Having such mechanical flexibility allows for manipulating these sensors in different conformations while still being able to obtain a viable signal (Figure 6). The resistance measurement was done using two alligator clips and a Fluke 179 True RMS Multimeter. The resulting form factor indicates that these can be easily adapted to technologies which can be embedded into clothing or worn as accessories. This is an avenue to be explored in a future work.

**Figure 7:**
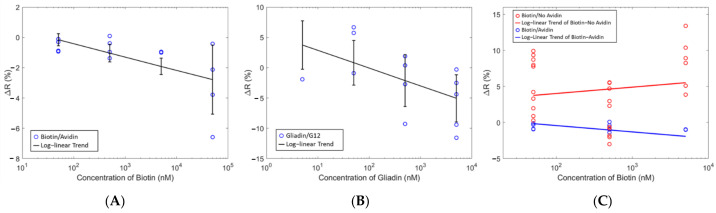
Change in resistance vs. concentration for (**A**) Biotin analyte with Avidin capture molecule (**B**) Gliadin analyte with G12 capture molecule (**C**) Biotin analyte with and without capture molecule. The circles represent the actual data, and the line is a linear fit to these data. The error bar represents the standard deviation from the predicted data from the linear fit.

The sensor measurements from Figure 7 show a log-linear correlation between the concentration and the change in resistance of the sensor upon the addition of analyte. At each concentration, approximately 4 identical measurements were performed which is indicated by multiple blue circles at one specific concentration. This change in resistance was proportional to the analyte concentration indicating the ability of the sensor to provide distinguishable signals for different analyte concentrations. Furthermore, similar responses were seen for the avidin/Biotin system and the G12/Gliadin with the only difference being the capture molecule (Figure 7B). Building upon these results, the next series of tests were performed to investigate the response from an analyte on a sensor with (specific binding) and without (nonspecific binding) the complementary capture molecule immobilized on the surface of the sensor. From Figure 7C, there was no longer a negative Δ*R* as seen with complementary analyte and capture molecules, but rather a positive Δ*R* was observed when no capture molecule was used. These results suggest that a negative Δ*R* is indicative of a specific binding event whereas a positive Δ*R* indicates a nonspecific binding event. This positive Δ*R* had a weak inverse correlation with the analyte concentration, which was not nearly as strong as in the case of the complementary binding pairs. Non-complementary analytes and capture molecules such as Gliadin and avidin respectively, also showed this behavior. Test measurements with pure PBS as the analyte were also done. There were no changes in the resistance which indicated that geometry or volume changes due to hydration did not influence the results.

A two-way ANOVA and Tukey’s pairwise test was performed to determine the statistical relationship of the data. ANOVA takes the mean and compares the variances between groups to determine if the observed effects is real or only due to chance. Tukey’s pairwise test was used in ANOVA to create the confidence intervals. These statistical methods considered Δ*R* for sensors, with and without Biotin, and the type of analyte. The analysis returned a statistically significant interaction effect between whether there is a protein bound to the sensor and the analyte used. These data suggests that the nonspecific binding response in our biosensors is the opposite of the specific binding response. Since similar results are seen in the data for Biotin and Gliadin, this also suggests that the signal is due to the presence/absence of complementary binding pairs and not individual effects from the bound protein to the analyte. However, further experimentation is necessary to support this observation. This behavior may be due to the electrical characteristics of the protein-capture molecule bonds that occur through specific binding events. This observation can be related to gas sensors made of conducting polymer-metal hybrids. By coupling metal particles to the conducting polymer films, the change in work function experienced by the metal in the presence of a gas can be translated into changes in the electrical characteristics of the polymer film [25,26]. The sensors in this work follow a similar architecture to the gas sensor previously mentioned which suggests that the interaction between the analyte and the capture molecule is modulating the electronic properties of the polymer film.

High surface area sensors are very important for improving sensitivity. The sensor architecture in this work has been compared to a sensor made with avidin-functionalized gold nanorod modified electrodes [14] which used the avidin-Biotin affinity couple for testing as well. Although the gold nanorod sensor has a higher surface area, the conducting polymer sensor made on the fabric has comparable detection limit of 1 ng/mL and 1 nM concentration of Biotin, respectively. The response time of the fabric sensor has also been reported to be <4 min. Since our sensor architecture is identical to the one in the report, we expect similar values for detection limit and response time.

**Figure 8:**
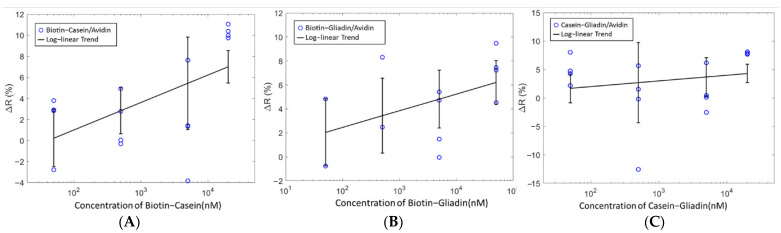
Change in resistance vs. concentration for mixed analytes all with Avidin capture molecule. (**A**) Biotin–Casein (**B**) Biotin–Gliadin, (**C**) Casein–Gliadin.

**Figure 9:**
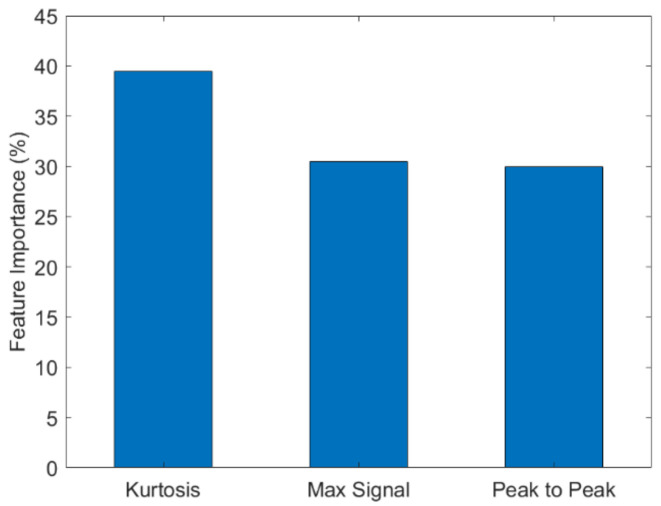
Feature importance based on Random Forest classification.

Machine learning is used to build an analytical model capable of identifying patterns to predict the presence of Biotin in mixed analytes. Mixed analyte solutions of Casein–Gliadin, Biotin–Gliadin and Biotin–Casein were tested with the sensor immobilized with avidin (Figure 8). Unlike the single analyte tests, the detection of Biotin cannot be inferred from a change in resistance only. Very poor classification accuracies were obtained when the change in resistance of the mixed analyte solutions were used as features in the machine-learning algorithms, as such other features were used. In Table 1, the results of the machine-learning classifiers used to predict Biotin in mixed analyte solutions are shown. The classification accuracies range from 67–75%. The highest accuracy for the prediction of Biotin was 75% obtained using RF. RF was also used to identify the feature with the highest contribution (Figure 9). Kurtosis yielded the highest contribution indicating that the sharpness of the peak is influenced by the presence of Biotin. Kurtosis is the measure of whether the probability distribution curve is heavy-tailed or light-tailed with respect to a normal distribution. A higher kurtosis value corresponds to large deviations from the mean of the curve and a lower value corresponds to values closer to the mean. When the analyte is added, the curve’s characteristics are different with the presence and absence of Biotin which would correspond to difference values for kurtosis. This makes kurtosis an important indicator for the detection of Biotin.

## 4. Conclusions

In this study, we developed a conducting polymer-based chemiresistive biosensor capable of label-free sensing of biomolecules. The focus was not the detection of any specific biomolecule but to show that this biosensing platform can detect distinct responses from specific and nonspecific binding events. When complementary analytes and capture molecules such as Biotin/avidin or Gliadin/G12 are measured, a negative Δ*R* across the sensor is seen when the analytes are added which we correlate with a specific binding event. On the other hand, when non-complementary analytes and captures molecules such as Gliadin/avidin and Biotin/G12 are measured a positive Δ*R* is observed which we correlate with nonspecific binding events. Since the same observation is made for two different sets of analytes and capture molecules, we believe that for this sensor the signal is based solely on whether the analytes and capture molecules are complementary. Furthermore, when the analyte is composed of a mixture of complementary and non-complementary molecules, the resistance is not a good measure (Figure 8) and machine-learning algorithms can be used to predict the presence of Biotin specifically, in mixed analytes. Our results show that random forests (RF) yielded the best prediction accuracy. Different feature extraction techniques were also used to obtain the highest possible accuracy. Based on the feature importance of random forest, kurtosis (Appendix A) was found to be the most important. Thus, using kurtosis as the feature and random forest as the algorithm we were able to predict the presence of Biotin with accuracies up to 75%.

## Figures and Tables

**Table 1:** Classification Accuracies.

ML Classifiers	Accuracy
Support Vector Machines	67%
Random Forest	75%
k-nearest neighbors	70%
Logistic Regression	67%

## Data Availability

Not Applicable.

## Appendix A

### Kurtosis

Kurtosis is the measure of whether the probability distribution curve is heavy-tailed or light-tailed with respect to a normal distribution.

For a univariate data *x*_1_, *x*_2_, *x*_3_, and *x*_4_, Kurtosis of the data is given by the Formula:

$$\frac{\sum_{i=1}^{N}{\left(x_{i}-\bar{x}\right)}^{4}/N}{S^{4}}$$

where $\bar{x}$ is the mean, *S* is the standard deviation and *N* is the number of points. Kurtosis is also known as the fourth moment of the distribution. A higher kurtosis value implies that the data is not concentrated around the mean and in general has larger presence of outliers. Kurtosis as a feature has been previously used in biological studies including activity recognition [27,28], developing imaging techniques [29,30]. All these previous studies imply the usefulness of using Kurtosis as a feature when dealing with signal data. We treat kurtosis as a feature of the signal feature because it is expected that the signal that corresponds to biotin will have a different distribution from the signal that does not have biotin presence. Moreover, the feature importance plot from the machine learning model justifies our selection of kurtosis as it describes which feature in the model was most important when doing the classification task.
